# Role of Blood Cell Indexes in Progresses to ESRD

**DOI:** 10.1007/s12291-024-01184-1

**Published:** 2024-02-13

**Authors:** Duong Thi Ngoc Lan, Donatella Coradduzza, Le Van An, Panagiotis Paliogiannis, Carla Chessa, Angelo Zinellu, Arduino A. Mangoni, Ciriaco Carru

**Affiliations:** 1 Department of General Internal Medicine and Endocrinology, Hue University of Medicine and Pharmacy, Hue University, Hue, Vietnam; 2 Department of Biomedical Sciences, University of Sassari, Sassari, Italy; 3 Department of Medical, Surgical, and Experimental Sciences, University of Sassari, 07100 Sassari, Italy; 4 Discipline of Clinical Pharmacology, College of Medicine and Public Health, Flinders University and Flinders Medical Centre, Bedford Park, SA 5042 Australia; 5 Department of Clinical Pharmacology, Flinders Medical Centre, Southern Adelaide Local Health Network, Bedford Park, SA 5042 Australia; 6 Control Quality Unit, Azienda-Ospedaliera Universitaria (AOU), 07100 Sassari, Italy

**Keywords:** Chronic kidney disease, Blood cell count, Neutrophil/lymphocyte ratio, Derived neutrophil/lymphocyte ratio, Renal progression, Transition to end-stage renal disease

## Abstract

Chronic kidney disease (CKD) is a complex health condition characterized by the gradual loss of renal function, often leading to end-stage renal disease (ESRD). It results from a combination of medical, environmental, and genetic factors. Predicting the rate of renal function decline and effectively managing the progression to ESRD is challenging in clinical practice. CKD assessment involves various indicators, including estimated glomerular filtration rate (eGFR), albuminuria levels, serum creatinine, and others. This study aimed to explore the predictive potential of specific blood cell indexes in forecasting further renal function decline and the transition from CKD stage 3–4 to ESRD. We assessed the following blood cell indexes in 377 CKD stage 3–4 patients: absolute neutrophil count (ANC), neutrophil/lymphocyte ratio (NLR), platelet/lymphocyte ratio (PLR), derived NLR (dNLR), mean platelet volume (MPV), aggregate index of systemic inflammation (AISI), and systemic inflammation index (SII). ANC, MPV, NLR, PLR, dNLR, and SII were found to independently predict a rapid decline in eGFR. Notably, NLR and dNLR demonstrated the highest sensitivity and specificity with cut-off values of 3.36 and 2.45, respectively (NLR: 88.6 and 81.7%; dNLR: 85.2 and 75.8%). The corresponding area under the ROC curve values were 0.877 (95% CI 0.837–0.918, *p* < 0.001) for NLR and 0.849 (95% CI 0.805–0.892, *p* < 0.001) for dNLR. However, none of the blood cell indexes independently predicted the transition to ESRD. The NLR and the dNLR exhibited the highest predictive capacity towards a rapid decline in renal function in CKD. No blood cell index, however, independently predicted the transition into ERSD.

## Introduction

The prevalence of chronic kidney disease (CKD) varies across different regions and is influenced by a range of factors, including population demographics, access to healthcare, and lifestyle choices [1]. Recognized as a global health concern by organizations such as the World Health Organization (WHO) and the International Society of Nephrology, CKD has shown a consistent increase in prevalence in many parts of the world. Globally, CKD affects more than 10% of the population to varying degrees, but the specific prevalence rates can differ significantly from one country or region to another [2]. Notably, low- and middle-income countries may experience higher prevalence rates due to challenges like limited access to healthcare services, the presence of infectious diseases, and environmental factors [3]. In the United States alone, CKD is a significant public health issue. According to estimates provided by the National Kidney Foundation and the Centers for Disease Control and Prevention (CDC), approximately 37 million adults in the U.S. were living with CKD, representing around 15% of the adult population. Ageing, diabetes, obesity, and hypertension are considered the main causes of CKD, and place a considerable financial burden in most advanced healthcare systems [2, 3]. Moreover, the number of deaths due to poor access to renal replacement therapy in developing countries remains unacceptably high [4].

Nephron loss triggers hypertrophy and hyperfiltration in remaining nephrons, and even with successful management of diabetes and hypertension, CKD often advances to end-stage renal disease (ESRD) affecting an estimated 4.902–7.083 million individuals worldwide. CKD has become a significant global health concern, with a prevalence of approximately 13.4%, impacting morbidity and mortality on a global scale. Its growth is primarily driven by factors such as diabetes, hypertension, obesity, and an aging population, though specific causes may vary regionally, including infections and environmental factors [4, 5]. However, significant challenges exist in clinical practice regarding the capacity to predict the temporal progression of CKD, preventing the institution of suitable preventive and management strategies. CKD is characterized by chronic low-grade inflammation, endothelial dysfunction, and platelet activation [6, 7]. The inflammatory state in CKD is associated with the release of cytokines and the production and activation of adhesion molecules that lead to T-cell adhesion and migration into the interstitium, with consequent stimulation of pro-fibrotic factors. Inflammation in CKD increases the risk of cardiovascular events, all-cause mortality, and its progression by contributing to the development of vascular calcifications and endothelial dysfunction [6]. Although several inflammatory markers have been investigated in CKD, such as CRP, erythrocyte sedimentation rate, interleukin (IL)-6, IL-8, IL-12, tumor necrosis factor (TNF)-alpha, and IL-33, their ability to predict disease progression is unclear. In recent years, routinely measured blood cell indexes in several diseases [8–12] as the neutrophil/lymphocyte ratio (NLR), platelet/lymphocyte ratio (PLR), monocyte/lymphocyte ratio (MLR), derived NLR (dNLR), systemic inflammation index (SII), and aggregate index of systemic inflammation (AISI) have been investigated as markers of inflammation in renal disease [13] and acute kidney injury after major abdominal surgery [14, 15]. However, their clinical significance has primarily been investigated in cancer and cardiovascular disease [16–20]. In this study we investigated the ability of these blood cell indexes to predict (a) the further decline in renal function in CKD and (b) the transition of CKD stage 3–4 to ESRD.

## Methods

We conducted a retrospective study of CKD stage 3–4 patients (defined as an e-GFR between 30–60 and 15–30 ml/min/1.73 m^2^, respectively); including patients with comorbidities such as type 2 diabetes, hypertension, coronary artery disease, and the elderly attending the nephrology outpatient clinic at the Hue Central Hospital in Viet Nam from October 2015 to October 2018. Sample size: the required sample sizes for estimating specificity concerning the pre-determined value of specificity ascertained by previously published data [21].

$$ n = \frac{{Z_{{\frac{\alpha }{2}}}^{2} \overset{\lower0.5em\hbox{$\smash{\scriptscriptstyle\frown}$}}{P} (1 - \overset{\lower0.5em\hbox{$\smash{\scriptscriptstyle\frown}$}}{P} )}}{{d^{2} }} $$

*P* = 0.69, is a pre-determined value of specificity from published data of Ismail Kocyigit et al. [22]; α = 0.05; marginal error d = 5%. Sample size n = 329.

Exclusion criteria were follow-up time <1 year, eGFR > 60 ml/min/1.73 m^2^, acute inflammation (defined by a WBC count>15,000/mm^3^ and/or a C-reactive protein (CRP) concentration > 50 mg/dL), hematological diseases, other chronic inflammatory processes (such as rheumatoid arthritis, ankylosing spondylitis, and gout), and treatment with immunosuppressive therapies. We also excluded CKD patients exhibiting changes in eGFR to values compatible with stages less than 3 or 4 during follow-up, leaving 377 patients with CKD stages 3–4 suitable for analysis.

Patients underwent clinical and biochemical profile assessments to evaluate CKD progression, including CRP, urea, and creatinine. NLR (neutrophil to lymphocyte ratio), PLR (platelet to lymphocyte ratio), MLR (monocyte to lymphocyte ratio), dNLR (derived NLR), RDW (Red Cell Distribution With), AISI (aggregate index of systemic inflammation, calculated by multiplying the counts of neutrophils, monocytes, and platelets and then dividing the product by the lymphocyte count), and SII (systemic inflammation index, calculated by (neutrophil counts × platelet counts)/lymphocyte counts) were assessed at baseline and then serially during outpatient visits. Smoking status, cause of CKD, type of antihypertensive drugs, and laboratory tests were also recorded during the first visit. The time point of the last outpatient visit within the 3 years was defined as the last assessment. ESRD is defined as an irreversible decline in kidney function, which requires dialysis or transplantation to improve the glomerular filtration rate (GFR) [23]. For patients who transitioned to ESRD before the last outpatient visit, the last assessment was defined as the time point of transition. Therefore, the time to transition to ESRD (months) was the time interval between the first visit and the time point of transition to ESRD.

Estimated glomerular filtration rate (eGFR) was calculated using the MDRD equation [24]. According to KDIGO (International Kidney Disease: Improving Global Outcomes) 2012 guideline classification CKD stage 3 and 4 were defined as an eGFR between 30–60 and 15–30 ml/min/1.73 m^2^, respectively [23]. eGFR decline, the primary endpoint of the study, was defined as rapid or slow if the eGFR reduction was either >= 5 mL/min/year or < 5 mL/min/year, respectively, according to the following formula: annual progression rate (mL/min/year) = baseline eGFR-last eGFR (ml/min/1.73 m^2^) ×12 months/number of months [25].

Transition to ESRD, or initiation of renal replacement therapy, was the secondary endpoint to categorize the patients. Patients with an eGFR less than 15 ml/min/1.73 m^2^ are considered to be in an advanced state of renal failure and are often in need of dialysis or kidney transplantation, whereas Patients with an eGFR between 15 and 60 ml/min/1.73 m^2^ may have a variety of renal conditions but are not yet considered to be in transition.

### Statistical Analysis

We expressed categorical data as percentage and number. Non-normally distributed variables were presented as median (range) and normally distributed variables as mean ± SD, as appropriate. Statistical significance was concluded when *p*-value < 0.05. We used the Chi-square test to assess between-group comparisons for nominal variables and the Independent Samples *T*-test to compare means between groups. Spearman’s rank correlation was used to determine correlations between paired variables. Variables showing associations with *p*-values <0.05 were included in logistic regression to determine their ability to independently predict the decline in eGFR and the transition to ESRD. We measured the ROC curve to determine the sensitivity, specificity, AUC, and cutoff values of predictive factors.

### Ethical Standards

Compliance with ethical standards and approved by Ethic Council in biomedical research of Hue University of Medicine and Pharmacy, Vietnam. Date of Approval: 6 January, 2018.

## Results

A total of 377 patients were included in the study (Table 1). There were no statistically significant differences in age, gender, BMI, smoking status, baseline serum creatinine, eGFR, type of antihypertensive drugs, blood cells (M, RDW, and PDW), and CRP concentrations between CKD patients with slow vs. rapid progression. There were significant differences in the duration of follow-up, causes of CKD, white blood cells (W), ANC(N), platelets (P), mean platelet volume (MPV), NLR, PLR, MLR, dNLR, SII, and AISI.

**Table 1:** Distribution of baseline characteristics according to eGFR decline

Baseline characteristics	All patients *n* = 377 Mean ± SD	Slow decline in eGFR (< 5 ml/min/1.73 m^2^) *n* = 289 Mean ± SD	Rapid decline in eGFR (≥ 5 ml/min/1.73 m^2^) *n* = 88 Mean ± SD	*p* value
Age	55.68 ± 15.98	55.84 ± 15.76	55.15 ± 16.76	0.721
Gender % (*n*)	100 (277)	76.7 (289)	23.3 (88)	0.074
Male	56.5 (213)	54 (156)	64.8 (57)	
Female	43.5 (164)	46 (133)	35.2 (31)	
BMI	22.75 ± 2.18	22.69 ± 2.23	22.97 ± 2.02	0.293
Smoking % (*n*)	100 (377)	76.7 (289)	23.3 (88)	0.967
Yes	30.5 (115)	30.4 (88)	30.7 (27)	
No	69.5 (262)	69.6 (201)	69.3 (61)	
Duration of follow-up (months)	22.16 ± 3.49	22.66 ± 3.21	20.52 ± 3.88	< 0.001
Baseline creatinine (mg/dL)	2.30 ± 0.62	2.3 ± 0.65	2.29 ± 0.53	0.879
eGFR (ml/min/1.73 m^2^)	30 ± 9.81	29.87 ± 10.08	30.4 ± 8.91	0.661
Type of antihypertensive drugs % (*n*)	100 (377)	76.7 (289)	23.3 (88)	0.841
ACEI	8.2 (31)	8 (23)	9.1 (31)	
ARB	16.7 (63)	16.3 (47)	18.2 (63)	
CCB	5.6 (21)	6.2 (18)	3.4 (21)	
ACEI + ARB	45.9 (173)	45 (130)	48.9 (173)	
ACEI/ARB + Diuretics	17.5 (66)	18 (52)	15.9 (66)	
ACEI + ARB + CCB	6.1 (23)	6.5 (19)	4.5 (23)	
Cause of CKD % (*n*)	100 (377)	76.7 (289)	23.3 (88)	0.001
Diabetes	31.8 (120)	26.3 (76)	50 (44)	
Glomerulonephritis	7.2 (27)	8 (23)	4.5 (4)	
Hypertension	42.2 (159)	45 (130)	33 (29)	
Polycystic kidney disease	2.7 (10)	2.4 (7)	3.4 (3)	
Unknown	16.2 (61)	18.3 (53)	9.1 (8)	
CRP (mg/L)	11.6 ± 9.14	11.4 ± 9.3	12.16 ± 8.72	0.547
WBC (cell/mm^3^)	7198 ± 1931	7073 ± 1916	7610 ± 1935	0.022
N (g/L)	4868 ± 1622	4587 ± 1539	5789 ± 1550	< 0.001
L (cell/mm^3^)	1827 ± 775	1980 ± 773	1325 ± 526	< 0.001
M (cell/mm^3^)	201 ± 91	197 ± 89	216 ± 94	0.084
P (cell/mm^3^)	245 ± 75	145 ± 71	195 ± 95	0.004
MPV (fL)	9.43 ± 1.04	9.13 ± 0.81	10.43 ± 1.09	< 0.001
RDW (%)	13.89 ± 5.94	13.44 ± 13.76	14.84 ± 12.04	0.086
PDW (%)	11.7 ± 4.12	11.66 ± 4.02	11.73 ± 4.4	0.897
NLR	3.15 ± 1.85	2.65 ± 1.59	4.8 ± 1.66	< 0.001
PLR	156 ± 80.1	251 ± 77	225 ± 64	< 0.001
MLR	0.12 ± 0.07	0.11 ± 0.56	0.17 ± 0.68	< 0.001
dNLR	1.26 ± 0.06	2.06 ± 1.08	3.47 ± 1.23	< 0.001
SII	762 ± 482	663 ± 421	1089 ± 528	< 0.001
AISI	154 ± 128	131 ± 111	230 ± 151	< 0.001

We run a correlation analysis between pre-specified variables and CKD progression rate. Variable showing associations with *p*-values < 0.05 were included in logistic regression to determine the independent predictive ability for the decline in eGFR and the transition to ESRD.

In univariate logistic regression analysis, the duration of follow-up, baseline eGFR, baseline WBC, baseline N, baseline L, baseline P, baseline MPV, baseline NLR, baseline PLR, baseline dNLR, baseline MLR, baseline SII, and baseline AISI were significantly associated with a rapid decline in eGFR. However, in multivariate analysis, only baseline N, baseline MPV, baseline NLR, baseline PLR, baseline dNLR, and baseline SII remained independent predictors of the rapid decline in eGFR (Table 2).

**Table 2:** Logistic regression analysis of rapid decline in eGFR

Baseline characteristic	Univariate				Multivariate			
	β	95%CI		*P* value	β	95%CI		*P* value
Duration of following (month)	− 0.235	− 0.219	− 0.089	< 0.001	− 0.036	− 0.069	0.022	0.305
Baseline eGFR (ml/min/1.73 m^2^)	0.164	0.015	0.061	0.001	− 0.029	− 0.032	0.019	0.601
Baseline WBC (cell/mm^3^)	0.134	0.000	0.000	0.009	− 0.363	− 0.001	0.000	0.302
Baseline N (cell/mm^3^)	0.376	0.000	0.001	< 0.001	0.930	0.000	0.002	0.007
Baseline L (cell/mm^3^)	− 0.446	− 0.002	− 0.001	< 0.001	− 0.221	− 0.002	0.000	0.148
Baseline PLT (× 10^9^/L)	− 0.133	− 0.007	0.001	0.01	0.033	− 0.004	0.006	0.667
Baseline MPV (fL)	0.626	1.198	1.545	< 0.001	0.514	0.960	1.292	< 0.001
Baseline NLR	0.551	0.576	0.786	< 0.001	0.379	0.126	0.810	0.007
Baseline PLR	0.305	0.006	0.011	< 0.001	0.460	0.005	0.022	0.003
Baseline dNLR	0.530	0.805	1.117	< 0.001	− 0.472	− 1.440	− 0.270	0.004
Baseline MLR	0.422	11.45	17.83	< 0.001	− 0.014	− 7.336	6.400	0.893
Baseline SII	0.431	0.02	0.02	< 0.001	− 0.509	− 0.004	0.000	0.014
Baseline AISI	0.336	0.005	0.008	< 0.001	− 0.016	− 0.069	0.022	0.902

**Fig. 1:**
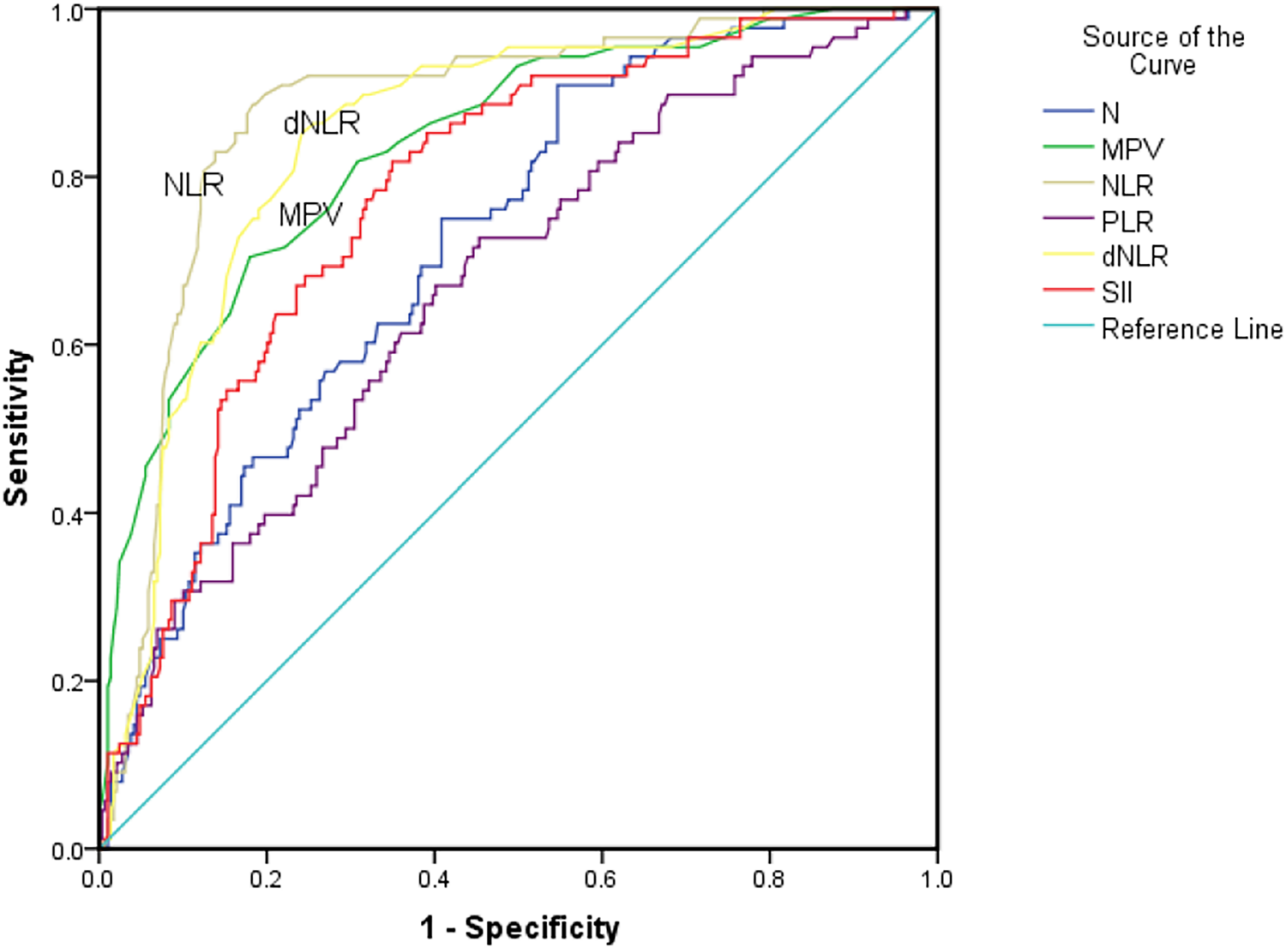
ROC analysis of high N, MPV, NLR, PLR, dNLR, SII as predictors of rapid decline in eGFR, N—neutrophil. MPV—mean platelet volume. NLR—neutrophil/lymphocyte ratio. PLR—platelet/lymphocyte ratio. dNLR—derived NLR. SII—systemic inflammation index. ROC—Receiver Operating Characteristics

The ability of N, MPV, NLR, PLR, dNLR, and SII to predict a rapid decline in eGFR was further assessed by ROC analysis (Fig. 1). The sensitivity and specificity of NLR and dNLR were higher than the other indexes, 88.6 and 81.7%; 85.2 and 75.8%, respectively, using a cutoff level of 3.36, and 2.45, respectively. AUC areas of NLR and dNLR were 0.877 (95% CI 0.837–0.918, *p* < 0.001) and 0.849 (95% CI 0.805–0.892, *p* < 0.001), respectively. The detailed data of other indexes are described in Table 3.

**Table 3:** ROC analysis of blood cell indexes

Index	AUC	Cutoff level	95%CI		*p* value	Sensitivity	Specificity
N	0.721	5450	0.665	0.778	< 0.001	52.3	76.1
MPV	0.836	9.85	0.788	0.884	< 0.001	70.5	82
NLR	0.877	3.36	0.837	0.918	< 0.001	88.6	81.7
PLR	0.671	137	0.608	0.734	< 0.001	72.7	54.7
dNLR	0.849	2.45	0.805	0.892	< 0.001	85.2	75.8
SII	0.779	673	0.727	0.830	< 0.001	81.8	65.1

**Fig. 2:**
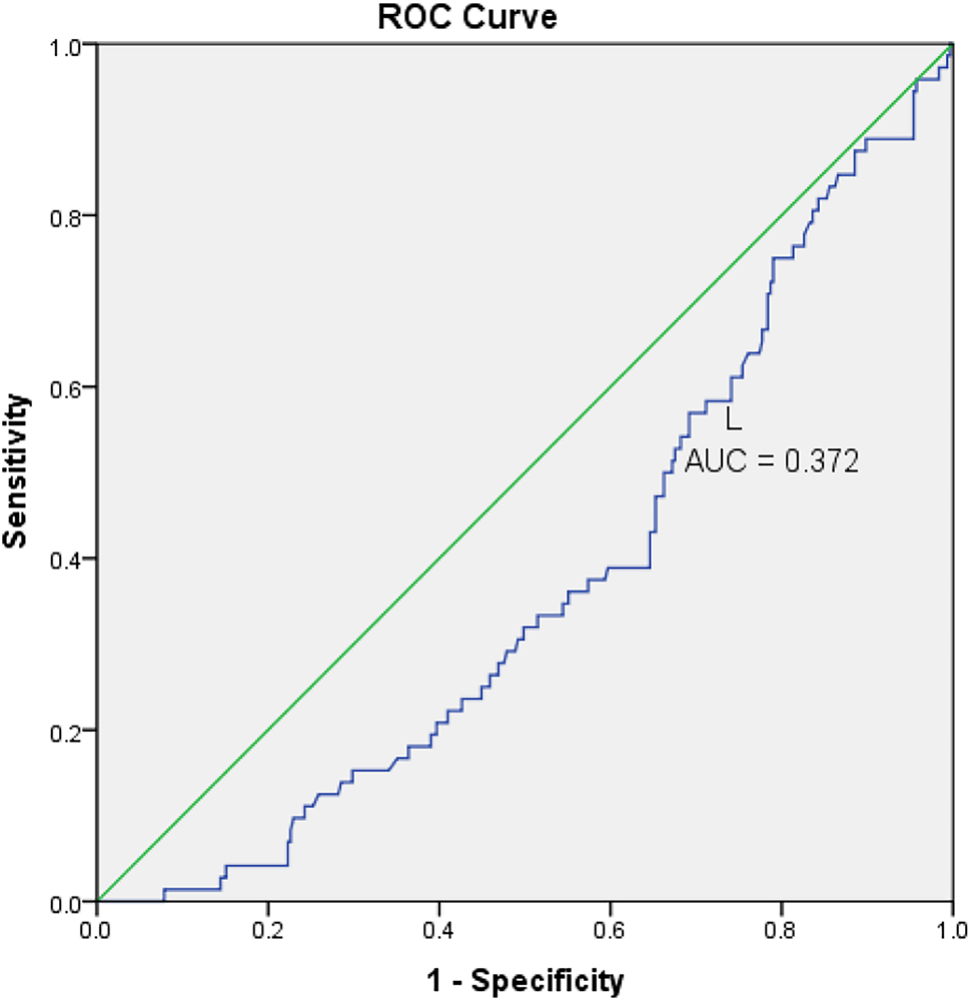
ROC analysis predictors of L transition to ESRD. L—lymphocyte. ROC—Receiver Operating Characteristics. ESRD—end stage renal disease

After a 3-year follow-up, 19.1% (72/377) CKD stage 3–4 transitioned to ESRD. There were no statistically significant differences in age, BMI, smoking status, duration of follow-up, type of antihypertensive drugs, causes of CKD, blood cells (W, N, M, P, MPV, RDW, and PDW), PLR and CPR concentrations between CKD patients with or without ESRD (Table 4). However, there were statistically significant differences in sex, baseline creatinine, eGFR, L, NLR, MLR, dNLR, SII, and AISI. Our results showed that patients transitioning to ESRD were also those with rapid eGFR deterioration (Fig. 2).

**Table 4:** The distribution of baseline characteristics according to transition to ESRD

Baseline characteristic	All patients *n* = 377 Mean ± SD	Non ESRD *n* = 305 Mean ± SD	ESRD *n* = 72 Mean ± SD	*p* value
Age	55.68 ± 15.98	55.55 ± 16.53	56.25 ± 13.46	0.738
Gender % (*n*)	100(377)	80.9 (305)	19.1 (72)	< 0.001
Male	56.5 (213)	62 (189)	33.3 (24)	
Female	43.5 (164)	38 (116)	66.7 (48)	
BMI	22.75 ± 2.18	22.68 ± 2.16	23.06 ± 2.23	0.181
Smoking % (*n*)	100(377)	80.9 (305)	19.1 (72)	0.09
Yes	30.5 (115)	32.5 (99)	77.8 (56)	
No	69.5 (262)	66.7 (206)	22.2 (16)	
Duration of follow-up (month)	22.16 ± 3.49	22.31 ± 3.45	21.51 ± 3.61	0.81
Baseline creatinine (mg/dL)	2.30 ± 0.62	2.15 ± 0.52	2.93 ± 0.6	< 0.001
eGFR (ml/min/1.73 m^2^)	30 ± 9.81	32.42 ± 9.21	19.72 ± 3.69	< 0.001
Kidney progression rate	100 (377)	80.9 (305)	19.1 (72)	< 0.001
Slow	76.7 (289)	88.3 (248)	56.9 (57)	
Rapid	23.3(88)	18.7 (41)	43.1 (31)	
Type of antihypertensive drugs % (*n*)	100 (377)	80.9 (305)	19.1 (72)	0.326
ACE	8.2 (31)	8.9 (27)	5.6 (4)	
ARB	16.7 (63)	16.1 (49)	19.4 (14)	
CCB	5.6 (21)	5.9 (18)	14.2 (3)	
ACEI + ARB	45.9 (173)	44.9 (137)	50 (36)	
ACEI /ARB + Diuretics	17.5 (66)	19 (58)	11.1 (8)	
ACEI + ARB + CCBs	6.1 (23)	5.2 (16)	9.7 (7)	
Cause of CKD % (*n*)	100 (377)	80.9 (305)	19.1 (72)	0.653
Diabetes	31.8 (120)	24.4 (92)	7.4 (28)	
Glomerulonephritis	7.2 (27)	5.8 (22)	1.3 (5)	
Hypertension	42.2 (159)	34.7 (131)	7.4 (28)	
Polycystic kidney disease	2.7 (10)	2.4 (9)	0.3 (1)	
Unknown	16.2 (61)	13.5 (51)	2.7 (10)	
CRP (mg/L)	11.60 ± 9.14	11.57 ± 9.17	11.73 ± 9.1	0.98
W (cell/mm^3^)	7198 ± 1931	7213 ± 1970	7138 ± 1776	0.769
N (cell/mm^3^)	4867 ± 4867	4808 ± 1628	5119 ± 1583	0.144
L (cell/mm^3^)	1827 ± 775	1894 ± 811	1544 ± 515	0.001
M (cell/mm^3^)	201 ± 91	201 ± 92	205 ± 85	0.714
P (cell/mm^3^)	245 ± 75	246 ± 73	239 ± 82	0.462
MPV (fL)	9.43 ± 1.04	9.4 ± 1.03	9.56 ± 1.09	0.259
RDW (%)	13.89 ± 5.94	13.63 ± 1.4	15 ± 13.3	0.081
PDW	11.7 ± 4.12	11.66 ± 3.83	11.75 ± 5.14	0.864
NLR	3.2 ± 1.84	3.03 ± 1.89	3.65 ± 1.58	0.011
NLR grade	100 (377)	80.9 (305)	19.1 (72)	*p* < 0.001
Low	63.5 (246)	70.2 (214)	44.4 (32)	
High	34.7 (131)	29.8 (91)	55.6 (40)	
PLR	156 ± 80.1	153 ± 81	170 ± 76	0.120
MLR	0.12 ± 0.07	0.12 ± 0.06	0.14 ± 0.07	0.004
dNLR	1.26 ± 0.06	2.31 ± 1.28	2.75 ± 1.1	0.007
SII	762 ± 482	738 ± 486	865 ± 455	0.045
AISI	154 ± 128	148 ± 121	182 ± 151	0.039

In univariate analysis, gender, baseline eGFR, kidney progression rate, baseline L, baseline P, baseline NLR, baseline NLR grade, baseline dNLR, baseline MLR, baseline SII, and baseline AISI were significantly associated with transition to ESRD. However, in multivariate analysis, only gender, baseline creatinine, kidney progression rate, and L had independent predictive roles in the transition to ESRD with *p*<0.001, *p*<0.001, *p* = 0.001 and *p* = 0.008, respectively) (Table 5).

**Table 5:** Logistic regression analysis of transition to ESRD

	Univariate				Multivariate			
	β	95%CI		*p* value	β	95%CI		*p* value
Gender	0.18	0.152	0.258	< 0.001	0.317	0.20	0.431	< 0.001
eGFR (ml/min/1.73 m^2^)	− 0.2	− 0.024	− 0.017	< 0.001	0.008	− 0.001	0.018	0.089
Kidney progression rate	0.21	0.119	0.302	< 0.001	0.154	0.060	0.249	0.001
L (cell/mm^3^)	− 9.012E − 005	0.000	0.000	0.001	− 0.005	0.000	0.000	0.008
NLR	0.028	0.006	0.049	0.011	− 0.050	− 0.114	0.015	0.131
NLR grade	0.175	0.093	0.257	< 0.001	0.082	− 0.034	0.198	0.164
dNLR	0.43	0.012	0.074	0.007	0.071	− 0.008	0.149	0.077
MLR	0.881	0.281	1.481	0.004	− 0.175	− 1.343	0.994	0.769
SII	8.450E − 005	0.000	0.000	0.045	0.000	0.000	0.000	0.197
AISI	0.000	0.000	0.001	0.039	0.000	0.000	0.001	0.183

## Discussion

This study showed that N, MPV, NLR, PLR, dNLR, and SII significantly predicted a rapid decline in eGFR, with both the NLR and the dNLR exhibiting the highest predictive capacity based on AUC values. The decline in eGFR was faster in patients with high NLR (cutoff level > 3.36) and dNLR (cutoff level > 2.45) group, results similar to those of Kocyigit's et al. [22]. Neutrophils release chemotactic substances that promote neutrophil migration to the kidney, worsening glomerular injury. These changes contribute to the fast progression of CKD. NLR provide information regarding inflammation patients in CKD [26], predicts the severity and prognosis of several pathological conditions chronic [27, 28], NLR and dNLR predict survival in breast cancer [29], urological [30], colorectal, and gastrointestinal tumors [31], high pre-treatment NLR (≥4) predicted a poor prognosis in lung cancer [32]. NLR could be used to predict cardiovascular endpoints in CKD patients in moderate to severe conditions associated with endothelial dysfunction, predicts all-cause mortality in geriatric patients with CKD [33]. However, its predictive role in the progression of CKD has not been comprehensively investigated. Our study demonstrates that dNLR independently predicts rapid CKD progression. In CKD patients, inflammation is associated with pro-coagulant and platelet-activating factors contributing to endothelial dysfunction [34]. PLR was shown to be superior to NLR in terms of inflammation in ESRD patients [35] and could independently predict all-cause mortality in hemodialysis patients [36]. SII has been shown to be a useful indicator [37]. In our study, both predicted a rapid decline in eGFR with sensitivity and specificity were inferior to that of NLR and dNLR. Also, CRP has a significant role in the rapid decline in eGFR supporting the potential advantage of using NLR and dNLR as predictors of eGFR decline in CKD stage 3–4. The ability of blood count cells to predict the transition to ESRD also was assessed by logistic regression and ROC analysis. We did not identify any useful blood cell index to predict the transition to ESRD. Several studies are in agreement with our study, which has shown that the percentage of patients transitioning into ESRD was not different between high and low-baseline NLR groups [38] and revealed that NLR is associated with the risk of ESRD in Chinese patients with stage 4 CKD [39, 40]. More research is warranted to clarify the role of the NLR and other inflammatory indexes in the progression of CKD. The main limitation of our study is the retrospective design and the lack of all patient data on the inflammation marker CRP (Table 6).

**Table 6:** L towards transition to ESRD

Index	AUC	95%CI		*p* value
L (cell/mm^3^)	0.372	0.306	0.438	0.001

L, lymphocyte; AUC, Area under the curve; Cl, confidence interval; ESRD, End stage renal disease

## Conclusion

In conclusion, our results suggest that the NLR and the dNLR have the highest predictive capacity of a rapid decline in eGFR and may represent useful markers for monitoring in CKD stage 3–4 in clinical practice because they are simple, inexpensive and easy to access.

## References

[1] Kovesdy CP. Epidemiology of chronic kidney disease: an update 2022. Kidney Int Suppl. 2022;12(1):7–11. 10.1016/j.kisu.2021.11.003 PMC9073222 35529086

[2] Collaborators GA. Global, regional, and national burden of diseases and injuries for adults 70 years and older: systematic analysis for the Global Burden of Disease 2019 Study. BMJ. 2022;376. 10.1136/bmj-2021-068208 PMC9316948 35273014

[3] Basto-Abreu A, Barrientos-Gutierrez T, Wade AN, Oliveira de Melo D, Semeão de Souza AS, Nunes BP, Perianayagam A, Tian M, Yan LL, Ghosh A, Miranda JJ. Multimorbidity matters in low and middle-income countries. J Multimorb Comorb. 2022; 12:26335565221106074. 10.1177/26335565221106074. PMID: 35734547; PMCID: PMC9208045. 10.1177/26335565221106074 PMC9208045 35734547

[4] Lv J-C, Zhang L-X. Prevalence and disease burden of chronic kidney disease. Renal Fibrosis Mech Therapies. 2019:3–15. 10.1007/978-981-13-8871-2_1 31399958

[5] Silverstein DM. Inflammation in chronic kidney disease: role in the progression of renal and cardiovascular disease. Pediatr Nephrol. 2009;24:1445–52. 19083024 10.1007/s00467-008-1046-0

[6] Liyanage T, Ninomiya T, Jha V, Neal B, Patrice HM, Okpechi I, Zhao MH, Lv J, Garg AX, Knight J, Rodgers A, Gallagher M, Kotwal S, Cass A, Perkovic V. Worldwide access to treatment for end-stage kidney disease: a systematic review. Lancet. 2015;385(9981):1975–82. 10.1016/S0140-6736(14)61601-9. (**Epub 2015 Mar 13 PMID: 25777665**). 25777665 10.1016/S0140-6736(14)61601-9

[7] Landray MJ, Wheeler DC, Lip GY, Newman DJ, Blann AD, McGlynn FJ, Ball S, Townend JN, Baigent C. Inflammation, endothelial dysfunction, and platelet activation in patients with chronic kidney disease: the chronic renal impairment in Birmingham (CRIB) study. Am J Kidney Dis. 2004;43(2):244–53. 14750089 10.1053/j.ajkd.2003.10.037

[8] Akchurin OM, Kaskel F. Update on inflammation in chronic kidney disease. Blood Purif. 2015;39(1–3):84–92. 25662331 10.1159/000368940

[9] Coradduzza D, Solinas T, Balzano F, Culeddu N, Rossi N, Cruciani S, Azara E, Maioli M, Zinellu A, De Miglio MR, Madonia M, Falchi M, Carru C. miRNAs as molecular biomarkers for prostate cancer. J Mol Diagn. 2022;24(11):1171–80. 10.1016/j.jmoldx.2022.05.005. (**Epub 2022 Jul 11 PMID: 35835374**). 35835374 10.1016/j.jmoldx.2022.05.005

[10] Coradduzza D, Ghironi A, Azara E, Culeddu N, Cruciani S, Zinellu A, Maioli M, De Miglio MR, Medici S, Fozza C, Carru C. Role of polyamines as biomarkers in lymphoma patients: a pilot study. Diagnostics (Basel). 2022;12(9):2151. 10.3390/diagnostics12092151. PMID: 36140552; PMCID: PMC9497571. 10.3390/diagnostics12092151 PMC9497571 36140552

[11] Coradduzza D, Arru C, Culeddu N, Congiargiu A, Azara EG, Scanu AM, Zinellu A, Muroni MR, Rallo V, Medici S, Carru C, Angius A, De Miglio MR. Quantitative metabolomics to explore the role of plasma polyamines in colorectal cancer. Int J Mol Sci. 2022;24(1):101. 10.3390/ijms24010101.PMID:36613539;PMCID:PMC9820724. 36613539 10.3390/ijms24010101 PMC9820724

[12] Coradduzza D, Solinas T, Azara E, Culeddu N, Cruciani S, Zinellu A, Medici S, Maioli M, Madonia M, Carru C. Plasma polyamine biomarker panels: agmatine in support of prostate cancer diagnosis. Biomolecules. 2022;12(4):514. 10.3390/biom12040514.PMID:35454104;PMCID:PMC9024899. 35454104 10.3390/biom12040514 PMC9024899

[13] Coradduzza D, Azara E, Medici S, Arru C, Solinas T, Madonia M, Zinellu A, Carru C. A preliminary study procedure for detection of polyamines in plasma samples as a potential diagnostic tool in prostate cancer. J Chromatogr B Analyt Technol Biomed Life Sci. 2021;1162: 122468. 10.1016/j.jchromb.2020.122468. (**Epub 2020 Nov 30 PMID: 33370684**). 33370684 10.1016/j.jchromb.2020.122468

[14] Ergin G, Değer SM, Köprü B, Derici Ü, ARINSOY ST. High neutrophil-to- lymphocyte ratio predicts acute allograft rejection in kidney transplantation: a retrospective study. Turkish J Med Sci. 2019;49(2):525–30. 10.3906/sag-1811-41 PMC7024429 30834734

[15] Gameiro J, Fonseca JA, Dias JM, Milho J, Rosa R, Jorge S, Lopes JA. Neutrophil, lymphocyte and platelet ratio as a predictor of postoperative acute kidney injury in major abdominal surgery. BMC Nephrol. 2018;19(1):320. 10.1186/s12882-018-1073-4.PMID:30419844;PMCID:PMC6231266. 30419844 10.1186/s12882-018-1073-4 PMC6231266

[16] Kolonko A, Dwulit T, Skrzypek M, Więcek A. Potential utility of neutrophil-to- lymphocyte, platelet-to-lymphocyte, and neutrophil, lymphocyte, and platelet ratios in differential diagnosis of kidney transplant acute rejection: a retrospective, propensity score matched analysis. Ann Transplant. 2022;27:e937239–41. 36536590 10.12659/AOT.937239 PMC9789674

[17] Catabay C, Obi Y, Streja E, Soohoo M, Park C, Rhee CM, Kovesdy CP, Hamano T, Kalantar-Zadeh K. Lymphocyte cell ratios and mortality among incident hemodialysis patients. Am J Nephrol. 2017;46(5):408–416. 10.1159/000484177. Epub 2017 Nov 7. PMID: 29130984; PMCID: PMC5777311. 10.1159/000484177 PMC5777311 29130984

[18] Yilmaz G, Sevinc C, Ustundag S, Yavuz YC, Hacıbekiroglu T, Hatipoglu E, Baysal M. The relationship between mean platelet volume and neutrophil/lymphocyte ratio with inflammation and proteinuria in chronic kidney disease. Saudi J Kidney Dis Transpl. 2017;28(1):90–4. 28098108 10.4103/1319-2442.198152

[19] Xiang F, Chen R, Cao X, Shen B, Liu Z, Tan X, Ding X, Zou J. Monocyte/lymphocyte ratio as a better predictor of cardiovascular and all-cause mortality in hemodialysis patients: A prospective cohort study. Hemodial Int. 2018;22(1):82–92. 10.1111/hdi.12549. (**Epub 2017 Apr 12 PMID: 28403540**). 28403540 10.1111/hdi.12549

[20] Solak Y, Yilmaz MI, Sonmez A, Saglam M, Cakir E, Unal HU, Gok M, Caglar K, Oguz Y, Yenicesu M, Karaman M, Ay SA, Gaipov A, Turk S, Vural A, Carrero JJ. Neutrophil to lymphocyte ratio independently predicts cardiovascular events in patients with chronic kidney disease. Clin Exp Nephrol. 2013;17(4):532–40. 10.1007/s10157-012-0728-x. (**Epub 2012 Nov 20 PMID: 23180042**). 23180042 10.1007/s10157-012-0728-x

[21] Tonyali S, Ceylan C, Yahsi S, Karakan MS. Does neutrophil to lymphocyte ratio demonstrate deterioration in renal function? Ren Fail. 2018;40(1):209–12. 29616601 10.1080/0886022X.2018.1455590 PMC6014370

[22] Kocyigit I, Eroglu E, Unal A, Sipahioglu MH, Tokgoz B, Oymak O, Utas C. Role of neutrophil/lymphocyte ratio in prediction of disease progression in patients with stage-4 chronic kidney disease. J Nephrol. 2013;26(2):358–65. 10.5301/jn.5000152. (**Epub 2012 May 8 PMID: 22573523**). 22573523 10.5301/jn.5000152

[23] Hajian-Tilaki K. Sample size estimation in diagnostic test studies of biomedical informatics. J Biomed Inform. 2014;48(2):193–204. 24582925 10.1016/j.jbi.2014.02.013

[24] KDIGO. Clinical practice guideline for the evaluation and management of chronic kidney disease. Offic J Int Soc Nephrol. 2012; 3(1).

[25] Levey AS, Bosch JP, Lewis JB, Greene T, Rogers N, Roth D. A more accurate method to estimate glomerular filtration rate from serum creatinine: a new prediction equation. Modification of Diet in Renal Disease Study Group. Ann Intern Med. 1999;130(6):461–70. 10.7326/0003-4819-130-6-199903160-00002 10075613

[26] Stevens PE, Levin A. Evaluation and management of chronic kidney disease: synopsis of the kidney disease: improving global outcomes 2012 clinical practice guideline. Ann Intern Med. 2013;158(11):825–30. 23732715 10.7326/0003-4819-158-11-201306040-00007

[27] Okyay GU, Inal S, Oneç K, Er RE, Paşaoğlu O, Paşaoğlu H, Derici U, Erten Y. Neutrophil to lymphocyte ratio in evaluation of inflammation in patients with chronic kidney disease. Ren Fail. 2013;35(1):29–36. 10.3109/0886022X.2012.734429. (**Epub 2012 Nov 1 PMID: 23113674**). 23113674 10.3109/0886022X.2012.734429

[28] Yao C, Liu X, Tang Z. Prognostic role of neutrophil-lymphocyte ratio and platelet- lymphocyte ratio for hospital mortality in patients with AECOPD. Int J Chron Obstruct Pulmon Dis. 2017;12:2285–90. 28814856 10.2147/COPD.S141760 PMC5546734

[29] Yang Z, Zhang Z, Lin F, Ren Y, Liu D, Zhong R, Liang Y. Comparisons of neutrophil-, monocyte-, eosinophil-, and basophil-lymphocyte ratios among various systemic autoimmune rheumatic diseases. APMIS. 2017;125(10):863–71. 10.1111/apm.12722. (**Epub 2017 Aug 2 PMID: 28766758**). 28766758 10.1111/apm.12722

[30] Krenn-Pilko S, Langsenlehner U, Stojakovic T, Pichler M, Gerger A, Kapp KS, Langsenlehner T. The elevated preoperative derived neutrophil-to-lymphocyte ratio predicts poor clinical outcome in breast cancer patients. Tumour Biol. 2016;37(1):361–8. 10.1007/s13277-015-3805-4. (**Epub 2015 Jul 29 PMID: 26219894**). 26219894 10.1007/s13277-015-3805-4

[31] Van Soest RJ, Templeton AJ, Vera-Badillo FE, Mercier F, Sonpavde G, Amir E, Tombal B, Rosenthal M, Eisenberger MA, Tannock IF, de Wit R. Neutrophil-to-lymphocyte ratio as a prognostic biomarker for men with metastatic castration-resistant prostate cancer receiving first-line chemotherapy: data from two randomized phase III trials. Ann Oncol. 2015;26(4):743–9. 10.1093/annonc/mdu569. (**Epub 2014 Dec 15 PMID: 25515657**). 25515657 10.1093/annonc/mdu569

[32] Absenger G, Szkandera J, Pichler M, Stotz M, Arminger F, Weissmueller M, Schaberl-Moser R, Samonigg H, Stojakovic T, Gerger A. A derived neutrophil to lymphocyte ratio predicts clinical outcome in stage II and III colon cancer patients. Br J Cancer. 2013;109(2):395–400. 10.1038/bjc.2013.346. Epub 2013 Jul 2. PMID: 23820252; PMCID: PMC3721404. 10.1038/bjc.2013.346 PMC3721404 23820252

[33] Yu Y, Qian L, Cui J. Value of neutrophil-to-lymphocyte ratio for predicting lung cancer prognosis: a meta-analysis of 7,219 patients. Molec Clinic Oncol. 2017;7(3):498–506. 10.3892/mco.2017.1342 PMC5547766 28811903

[34] Tatar E, Mirili C, Isikyakar T, Yaprak M, Guvercin G, Ozay E, Asci G. The association of neutrophil/lymphocyte ratio and platelet/lymphocyte ratio with clinical outcomes in geriatric patients with stage 3–5 chronic kidney disease. Acta Clin Belg. 2016;71(4):221–6. 10.1080/17843286.2016.1159797. (**Epub 2016 May 20 PMID: 27309205**). 27309205 10.1080/17843286.2016.1159797

[35] Conway DS, Pearce LA, Chin BS, Hart RG, Lip GY. Plasma von Willebrand factor and soluble p-selectin as indices of endothelial damage and platelet activation in 1321 patients with nonvalvular atrial fibrillation: relationship to stroke risk factors. Circulation. 2002;106(15):1962–7. 12370220 10.1161/01.cir.0000033220.97592.9a

[36] Turkmen K, Erdur FM, Ozcicek F, Ozcicek A, Akbas EM, Ozbicer A, Demirtas L, Turk S, Tonbul HZ. Platelet-to-lymphocyte ratio better predicts inflammation than neutrophil-to-lymphocyte ratio in end-stage renal disease patients. Hemodial Int. 2013;17(3):391–6. 10.1111/hdi.12040. (**Epub 2013 Mar 24 PMID: 23522328**). 23522328 10.1111/hdi.12040

[37] Yaprak M, Turan MN, Dayanan R, Akın S, Değirmen E, Yıldırım M, Turgut F. Platelet-to-lymphocyte ratio predicts mortality better than neutrophil-to-lymphocyte ratio in hemodialysis patients. Int Urol Nephrol. 2016;48(8):1343–8. 10.1007/s11255-016-1301-4. (**Epub 2016 Apr 27 PMID: 27118565**). 27118565 10.1007/s11255-016-1301-4

[38] Feng JF, Chen S, Yang X. Systemic immune-inflammation index (SII) is a useful prognostic indicator for patients with squamous cell carcinoma of the esophagus. Medicine. 2017;96(4): e5886. 28121932 10.1097/MD.0000000000005886 PMC5287956

[39] Altunoren O, Akkus G, Sezal DT, Ciftcioglu M, Guzel FB, Isiktas S, Torun GI, Uyan M, Sokmen MF, Sevim HA, Sarısık FN, Senel ME, Erken E, Gungor O. Does neutrophyl to lymphocyte ratio really predict chronic kidney disease progression? Int Urol Nephrol. 2019;51(1):129–37. 10.1007/s11255-018-1994-7. (**Epub 2018 Oct 1 PMID: 30276600**). 30276600 10.1007/s11255-018-1994-7

[40] Yuan Q, Wang J, Peng Z, Zhou Q, Xiao X, Xie Y, Wang W, Huang L, Tang W, Sun D, Zhang L, Wang F, Zhao MH, Tao L, He K, Xu H; C-STRIDE study group. Neutrophil-to-lymphocyte ratio and incident end-stage renal disease in Chinese patients with chronic kidney disease: results from the Chinese Cohort Study of Chronic Kidney Disease (C-STRIDE). J Transl Med. 2019;17(1):86. 10.1186/s12967-019-1808-4. PMID: 30876475; PMCID: PMC6420746. 10.1186/s12967-019-1808-4 PMC6420746 30876475

